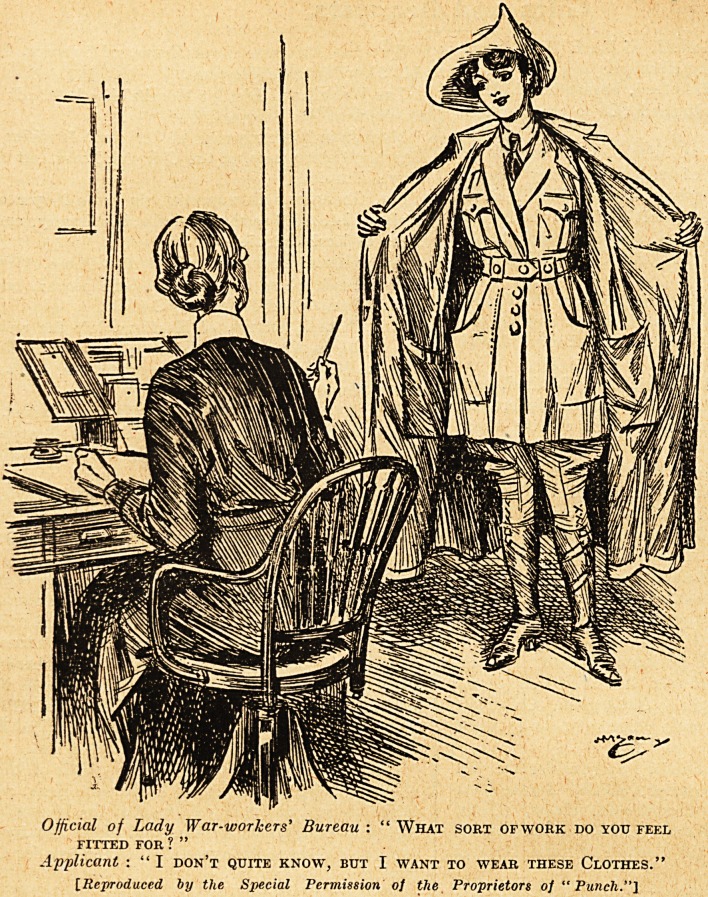# Round the Hospitals

**Published:** 1917-07-21

**Authors:** 


### ROUND THE HOSPITALS.

THE draft Royal Warrant, issued by the Ministry of Pensions and published in the *Morning Post* of the 16th instant, contains further provisions for nurses, which follow generally those for officers. The terms of the Warrant declare that the provision is on a very much more liberal scale than the existing regulations allow. Thus a staff nurse totally disabled through war service will receive £100 a year, as compared with £40 at present, and there are special grants for hospital and sanatorium treatment, for training, for medical expenses, and for constant attendance. A nurse disabled from unfitness not attributed to or aggravated by service may receive a gratuity which in exceptional circumstances may amount to £200. The present regulations allow only three months' pay. Three schedules showing the proposed scales of pensions are appended to the Warrant. The third schedule, dealing with pensions to disabled nurses, gives eight degrees of disablement, in which the sums payable vary from (1) 100 per cent. to (8) 20 per cent. In those cases where a matron is not entitled to service pension the disablement pension for Principal Matron or Matron-in-Chief varies from £175 in the first degree to £35 in the eighth degree. In the case of each matron it varies from £125 to £25. And in the case of a staff nurse or sister the disablement pension ranges from £100 to £20. When these officers are entitled to service pensions they also may receive an addition of from £75 in the first degree to £15 in the eighth degree. It is proposed that the new Warrant shall have effect from April 1, 1917. In the case of members of the Nursing Service whose claims to retired pay, pensions, or gratuities have been dealt with or arose under previous Warrants, the terms of this new Warrant may, if more beneficial to them, be applied with retrospective effect from April 1, 1917, on such dates as the Minister of Pensions may find it practicable and convenient to reassess these claims in accordance with instructions to be issued by him.

THE Belfast members of the Irish Board of the College of Nursing, Ltd., have invited Miss Matheson to deliver an address in explanation of the purposes and advantages of the College to a meeting of Belfast nurses to be held at the Medical Institute, College Square North, Belfast, at 8 P.M. on Thursday, July 26.

MISS JESSIE CAMPBELL, after twenty-three years' active work, has resigned the matronship of the Victoria Infirmary, Glasgow, from circumstances over which she had no control. Miss Campbell began as a probationer in the Victoria Infirmary, Glasgow, in 1893. She duly received her certificate in 1897, when the governors appointed her matron of the convalescent home in connection with the Infirmary at Brooksby, Largs, Ayrshire. Here Miss Campbell remained for three and a half years. She then returned to the Victoria Infirmary as assistant matron under Miss Macfarland. On the latter's resignation in October 1910, the board of the Victoria Infirmary elected Miss Campbell to the post of matron, an office she has filled ever since. Miss Campbell has thus completed twenty-three years' work in connection with the Victoria Infirmary, Glasgow, and has it in contemplation to take a prolonged holiday before again devoting her energies to the business of her profession. On leaving Glasgow, Miss Campbell was presented with a gold watch and thirty guineas in a Treasury-note case by the ladies of the Dorcas Society, a body in which she has always taken a practical and sympathetic interest. Mrs. Maitland Ramsay, in making the presentation, expressed sincere regret at losing Miss Campbell, who was one of their oldest friends and possessed the sincere regard of all members of the Dorcas Society. Mrs. Ramsay described Miss Campbell as a woman of deeds rather than words, who not only before the war, but during the last two and a half years especially, had stood as one "who helped by example, and her devotion to duty." Mrs. Maitland Ramsay assured Miss Campbell that the affectionate regard of her friends would go with her in whatever labours she might take up.

RECENTLY the honorary staff of the Worcester General Infirmary presented Miss Herbert, the late matron of that institution, with a handsome silver tea-service, her acceptance of which was invited as a small recognition of her long and valuable services to the infirmary and of the cordial relations which always existed between Miss Herbert and the honorary staff.

WE are asked to call attention again to the Imperial Nurses' Club, opened in November last at 137 Ebury Street, London, S.W. 1, by H.R.H. Princess Beatrice, its patroness. It now numbers three hundred members, and has proved a popular and helpful resort to those nurses who have no home in London and feel the need of somewhere to spend their free hours. Grateful letters of appreciation have been received from nurses whose homes are oversea or in the provinces, and whose duties have called them to France, Belgium, or elsewhere. Some nurses on the staff of military and civil hospitals, the Vice-Presidents state in their letter of appeal, have received refreshment in body and mind by sleeping at the Imperial Nurses' Club in restful surroundings on their off-duty nights. The Vice-Presidents appeal for the sum of £4,000 "to enable the Club to continue and "enlarge its invaluable work, to place it firmly on "its feet and make it self-supporting." Contributions can be sent to the Treasurers, Martin's Bank, 68 Lombard Street, E.C. 3, or to the Honorary Secretary, Miss C. H. Mayers, at the Club House, 137 Ebury Street, London, S.W. 1.

WE have pleasure in calling attention to the excellent work accomplished in its third year by the Ladies' Auxiliary Committee of the Royal Salop Infirmary, Shrewsbury. The Linen League for

*Official of Lady War-workers' Bureau*: "WHAT SORT OF WORK DO YOU FEEL FITTED FOR?"
*Applicant*: "I DON'T QUITE KNOW, BUT I WANT TO WEAR THESE CLOTHES."
[*Reproduced by the Special Permission of the Proprietors of "Punch."*]

**THAT UNIFORM ONCE MORE!**

LAST week in dealing with the uniform of the British nurse we mentioned as a fact that the question of uniforms seems to be uppermost with the majority of V.A.D.s. *Punch*, with that wonderful prescience and close touch with passing moods in classes of the community, now possessed by the ablest members of its editorial staff, has emphasised this aspect of uniforms, and applied it with telling force to the case of certain ladies ambitious of becoming war-workers in these days. Our readers will welcome the admirable illustration from *Punch*, which tells its own story.

some time has supplied all the linen to the infirmary and Nurses' Home, and has now undertaken to supply mackintosh sheets, to repair the mattresses, and to provide everything which is scheduled in the infirmary accounts as linen. The workers for the League, including the vice-presidents, number 409, who obtained £135 in subscriptions and provided during the year 896 articles and forty-four special gifts. The Ladies' Auxiliary has a Hamper Committee which organises pound days, and during the last year supplied more than half the potatoes and all other vegetables, fruit, and eggs required by the Salop Infirmary. The value of the gifts received, other than vegetables and fruit, is estimated at over £540, being an increase of £123 over the previous year. The committee justly express appreciation for the gifts mentioned, for they prove a real help not only in the saving of expenditure, but also in providing variation in diet and fresh dairy, farm, and garden produce for the in-patients and the infirmary. The Ladies' Auxiliary Committee is represented on the board of the hospital by three lady directors, to whose admirable work the report bears testimony. Taken altogether, these facts illustrate once more the invaluable work at present being done on behalf of our county hospitals by Ladies' Associations and Auxiliary Committees. Indeed, the inhabitants of Salop have good reason to be proud of their women for the valuable personal and material services they have rendered to the Shrewsbury Infirmary, its patients and managers.

---

IN response to many inquiries, arrangements have been made whereby mounted copies of the photograph of the historic group comprising Mr. J. G. Wainwright, the late Treasurer, taken with Miss Lloyd Still, the matron, and the sisters of St. Thomas's Hospital, published in our issue of July 7 last, p. 279, can be obtained from the publishers of THE HOSPITAL, 28 and 29 Southampton Street, Strand, London, W.C. 2, price 3s. 6d. each, or 3s. 9d. post free.

## Figures and Tables

**Figure f1:**